# Multiple mechanisms sustain a plant-animal facilitation on a coastal ecotone

**DOI:** 10.1038/srep08612

**Published:** 2015-02-27

**Authors:** Qiang He, Baoshan Cui

**Affiliations:** 1 School of Environment, State Key Laboratory of Water Environment Simulation, Beijing Normal University, Beijing 100875, China

## Abstract

Theory suggests that species distributions are expanded by positive species interactions, but the importance of facilitation in expanding species distributions at physiological range limits has not been widely recognized. We investigated the effects of the nurse shrub *Tamarix chinensis* on the crab *Helice tientsinensis* on the terrestrial borders of salt marshes, a typical coastal ecotone, where *Tamarix* and *Helice* were on their lower and upper elevational distribution edges, respectively. Crab burrows were abundant under *Tamarix*, but were absent in open areas between *Tamarix*. Removing *Tamarix* decreased associated crab burrows with time, while simulating *Tamarix* in open areas by shading, excluding predators, and adding *Tamarix* branches as crab food, increased crab burrows. Measurements of soil and microclimate factors showed that removing *Tamarix* increased abiotic stress, while simulating *Tamarix* by shading decreased abiotic stress. Survival of tethered crabs was high only when protected from desiccation and predation. Thus, by alleviating abiotic and biotic stresses, as well as by food provision, *Tamarix* expanded the upper intertidal distribution of *Helice*. Our study provides clear evidence for the importance of facilitation in expanding species distributions at their range limits, and suggests that facilitation is a crucial biological force maintaining the ecotones between ecosystems.

Many species are changing their distributions in response to climate change, such as elevational upper limits of alpine^1^ and intertidal species^2^. Predicting species distribution changes, however, needs to incorporate species interactions that can alter species individualistic responses to environmental change^3^,^4^. Negative and positive species interactions both affect community dynamics, and their relative importance changes with environmental stress, with facilitation more prevalent in stressful than in benign environments^5^,^6^. Although facilitation is suggested to expand the distributions and realized niche of species^7^,^8^, the importance of facilitation in expanding species distributions at their physiological range limits remains not widely recognized or tested in the field. An ideal habitat to examine this role of facilitation is ecotone, which is the transitional area between different ecosystems. Ecotones, such as terrestrial borders of salt marshes in the intertidal^9^,^10^,^11^ and alpine treelines^12^,^13^, are species distribution edges where species adapted to their native ecosystems face environmental stress of their tolerance limits. Here, we examine facilitation by a nurse plant on distribution expansion of a crab on the terrestrial borders of salt marshes.

Although facilitation theory applies to both plants and animals, it has only been extensively investigated in plant communities^6^. Plant-plant facilitation is widely known to be owing to alleviation of both abiotic (e.g. water, salinity) and biotic (e.g. herbivory) stresses, while plant-animal facilitation is often attributed to alleviation of abiotic stress, particularly in physically stressful intertidal ecosystems where consumer pressure is traditionally thought to be low^14^. For example, plant-animal facilitations on high rocky intertidal shores are often attributed to alleviation of desiccation stress^15^, rather than associational defense, because on rocky shores predation is often high only in the low intertidal. Similarly, plant-animal facilitations in salt marshes were also attributed to the alleviation of abiotic stress^15^,^16^,^17^. This is contrary to studies of subtidal ecosystems, such as seagrass beds, kelp forests, and coral reefs, where predation has long been known to drive plant-animal facilitations^18^,^19^,^20^. Although predation in intertidal habitats can be high, particularly by mobile species such as avian predators^17^,^21^, it is generally ignored in plant-animal facilitation investigations. Furthermore, previous studies often attribute plant-animal facilitation to alleviation of either abiotic (e.g. water, salinity) or biotic (e.g. herbivory) stresses, which are not mutually exclusive. Field investigations of whether these two mechanisms of facilitation can co-exist remain very few (but see Refs. 22, 23), however. Additionally, although plants may be a food resource for their interacting animals, few studies have disentangled the roles of food provision vs. non-trophic facilitation by plants on animals^24^.

We report two-year field experiments examining the interactions between the shrub *Tamarix chinensis* and the crab *Helice tientsinensis* on the terrestrial borders of two northern Chinese salt marshes (see Methods). These borders are a typical coastal ecotone^10^, which is flooded only during spring and storm tides, and are dry and hypersaline, leading to extreme thermal and desiccation stress for marine animals^9^,^16^,^17^. *Tamarix* is a nurse shrub distributed in coastal uplands without tides. Although *Tamarix* persists on the terrestrial borders, it is absent from lower marshes^9^. In contrast, *Helice* is a crab found mainly in intertidal marsh habitats, and is absent from coastal uplands^25^,^26^. On the terrestrial borders, both *Tamarix* and *Helice* are on their habitat edges. *Helice* burrows are abundant under *Tamarix*, but absent in open areas between *Tamarix*.

We conducted *Tamarix* removal, *Tamarix* simulation and crab tethering experiments to test the following hypotheses: (1) *Tamarix* is a critical factor in expanding *Helice* distributions on the physiological range limits, and (2) the facilitative effects of *Tamarix* on *Helice* are due to alleviation of both abiotic and biotic stresses (i.e. associational defense against predation).

## Results

**Figure 1:**
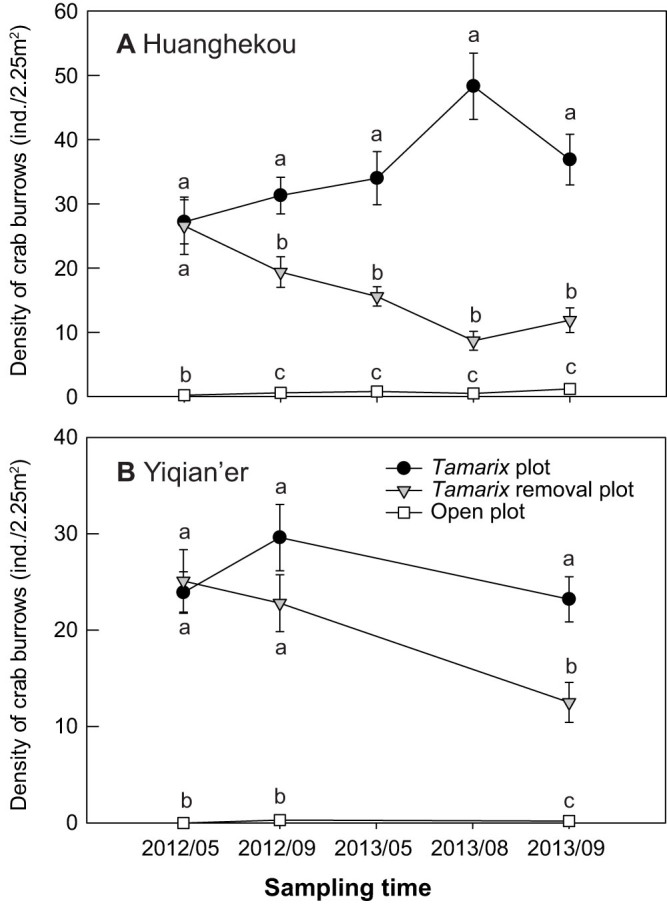
*Tamarix* removal experiment: density of crab burrows in each treatment at Huanghekou (A) and Yiqian'er (B). Data are means ± SE (*n* = 10). Within each sampling time, bars sharing a letter are not significantly different from one another (*P* > 0.05; Tukey HSD multiple comparisons).

**Figure 2:**
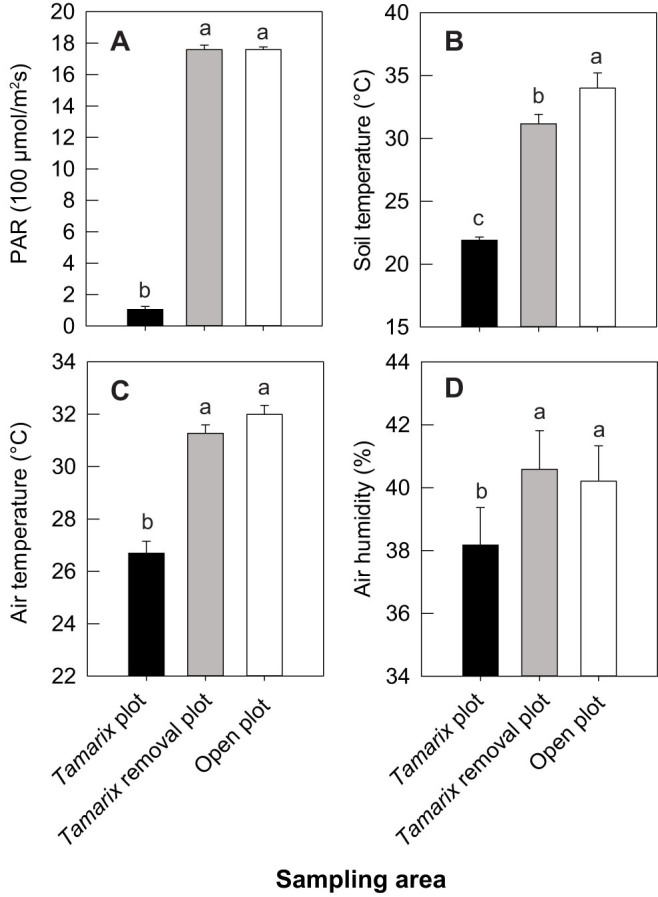
*Tamarix* removal experiment: microclimate factors in each treatment at Huanghekou. (A), Photosynthetically active radiation (PAR); (B), soil temperature; (C), air temperature; and (D), air humidity. Data are means + SE (*n* = 10). Bars sharing a letter are not significantly different from one another (*P* > 0.05).

**Figure 3:**
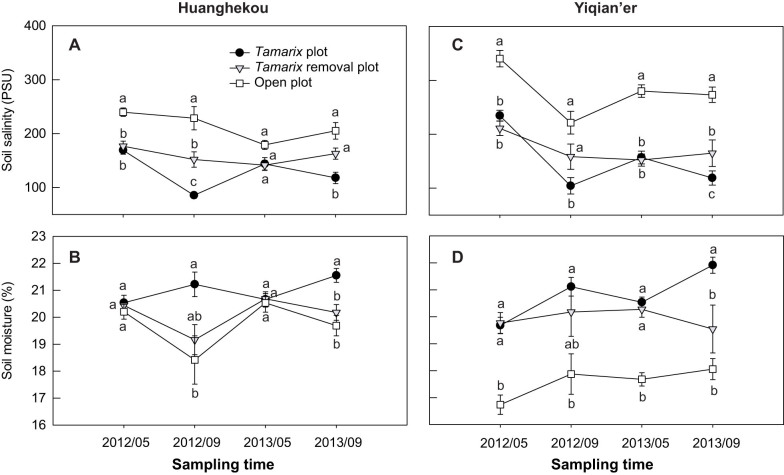
*Tamarix* removal experiment: soil condition in each treatment at Huanghekou and Yiqian'er. (A) and (C), Soil salinity; (B) and (D), soil moisture. Data are means ± SE (*n* = 10). Within each sampling time, bars sharing a letter are not significantly different from one another (*P* > 0.05).

At both sites, crab burrows were generally absent in open areas, but were dense under *Tamarix* (Fig. 1). At the beginning of the *Tamarix* removal experiment, there was no significant difference in burrow density between *Tamarix* and *Tamarix* removal plots (*P* > 0.05; Fig. 1). Removing *Tamarix* led to gradual decreases in crab burrows, and decreased by >50% after two years (Fig. 1). *Tamarix* removal significantly elevated solar radiation, soil temperature, and air temperature and humidity to levels similar to open plots but significantly different from *Tamarix* plots (Fig. 2). Soil salinity was significantly higher in open than in *Tamarix* plots in September, but not in May (Fig. 3). In contrast, soil moisture was significantly lower in open than in *Tamarix* plots in September, but not in May (Fig. 3). At the beginning of the experiment, there was no difference in soil salinity and moisture between in *Tamarix* and removal plots (Fig. 3). Removing *Tamarix* increased soil salinity and decreased soil moisture to levels similar to open plots but signficantly different from *Tamarix* plots (Fig. 3).

**Figure 4:**
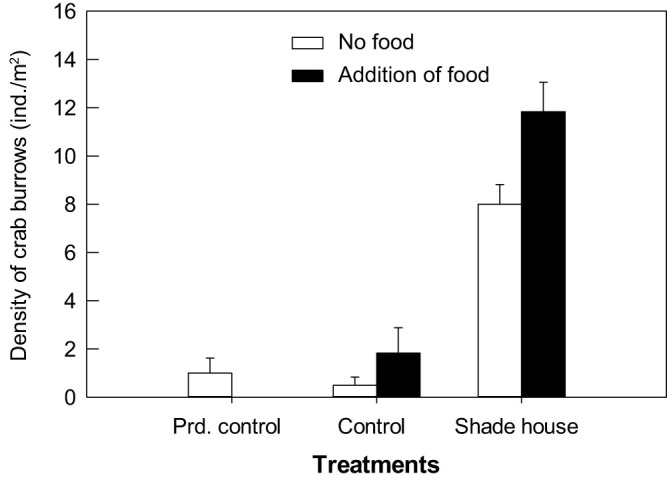
*Tamarix* simulation experiment: density of crab burrows in each treatment. Data are means + SE (*n* = 6). Prd. control indicates procedural control.

Simulation of *Tamarix*'s shading and predation-protection using shade houses significantly increased crab burrows in open areas (Fig. 4; *F*_1,20_ = 90.8, *P* < 0.0001). Addition of *Tamarix* as crab food also significantly increased crab burrows in open areas (Fig. 4; *F*_1,20_ = 7.02, *P* = 0.015). There were no significant interactive effects of shade house and food addition on crab burrows (Fig. 4; *F*_1,20_ = 0.04, *P* = 0.84). Differences were also not found in the number of crab burrows between procedural control and control treatments (df = 1, χ^2^ = 0.29, *P* = 0.59). Shading open areas with shade houses significantly decreased solar radiation, soil and air temperature, air humidity and soil salinity, and increased soil moisture, all of which became similar to *Tamarix* plots but different from open plots (*P* < 0.05, Supplementary Fig. S1; also see Figs. 2 and 3).

**Figure 5:**
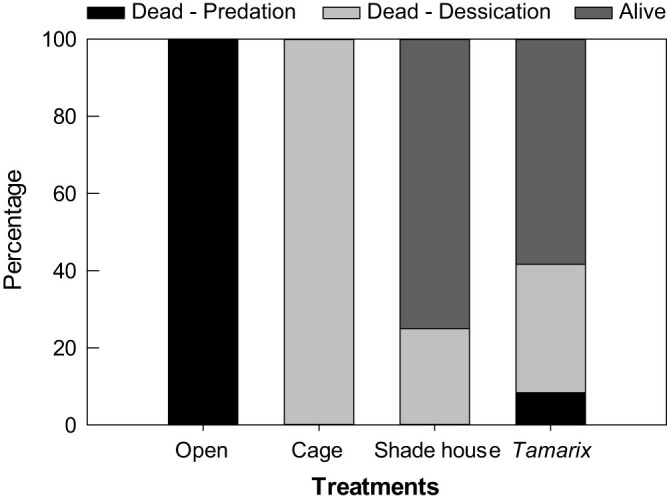
Crab tethering experiment: proportion of dead (by desiccation or predation) and live crabs in each treatment.

Survivorship of crabs tethered in *Tamarix* plots was 60% (Fig. 5), significantly higher than in open plots (*P* = 0.0046) and cage plots (*P* = 0.0046), but not significantly different from shade-house plots (*P* = 0.67). All crabs in open and cage plots were dead. All dead crabs in cage plots (as well as in *Tamarix* and shade-house plots) were intact, indicating that the death was mainly caused by abiotic stress. In contrast, all dead crabs in open plots had missing body parts and crushed carapaces, which, along with the presence of bird feces and footprints, indicates that predation caused the death.

## Discussion

Our results reveal that *Helice* is dependent on *Tamarix* on the terrestrial borders of salt marshes, and that this dependence is not only because *Tamarix* is a food resource for *Helice*, but also strongly due to non-trophic facilitation. Thus, in the coastal ecotone, facilitation expands the landward distribution of marsh crabs. Our work provides an unambiguous demonstration for the critical role of facilitation in mediating species distributions in natural communities, and in maintaining ecotones.

### Facilitation and species range expansion at physiological range limits

Our results show that thermal and desiccation stresses on the terrestrial borders of salt marshes are extreme and lethal to *Helice* (tethered *Helice* without shading all died, and *Helice* burrows are absent in open areas). Where *Tamarix* occurs, shading by its canopy retains soil moisture and decreases thermal and desiccation stress, creating microhabitats that are physically suitable to *Helice*. Thermal and desiccation stresses are the major abiotic factors limiting the distribution of marine animals in the upper intertidal, and alleviation of these stresses by shading has commonly been found to drive their facilitative interactions^15^,^16^. Although it has been argued that facilitation should collapse with extreme stress due to diminished effects of neighbors, or switch to competition for limiting resources (reviewed in Ref. 8), our results provide no support for these arguments (also see Ref. 27). The two species studied in our work are on different trophic levels and do not compete for limiting resources anyway. Although adult *Tamarix* persist on the terrestrial borders of salt marshes, abiotic stress such as salinity strictly limits its growth and regeneration^9^. This suggests that even in habitats where benefactor species themselves are severely limited^28^, facilitation can still function as a structuring force of communities.

Our work provides a straightforward example of how facilitation expands species' realized niche at their vertical distribution limits. Facilitation by *Tamarix* drives the expansion of the distribution of *Helice* from marshes at low elevations to the upper terrestrial border. Without *Tamarix*, the distribution of *Helice* would retract to lower elevations that are more frequently flooded. Facilitation has also been shown to expand the high intertidal borders of algae and invertebrates^15^, the low intertidal limits of marsh plants^9^,^29^, the arid borders of plants in dry habitats^8^,^30^, and the landward expansion of mangroves^31^. Our work, together with these studies, support the hypothesis that including facilitation in niche theory leads to species realized niches being larger than their fundamental niches^7^,^8^.

### Biotic drivers of plant-animal facilitation in physically stressful habitats

Our results also show that in addition to alleviation of abiotic stress, *Tamarix*'s associational defense against predation is a mechanism of facilitation of *Helice*. Many seabirds such as terns are abundant at our study sites and feed on *Helice*. It is also known that some seabirds prefer to feed in unvegetated habitats, possibly due to the ease of walking and attacking without vegetation (see Ref. 17). The existence of *Tamarix* on the largely bare terrestrial borders thus reduces *Helice'*s risk of predation. A previous study also suggested that the facilitation effects of vegetation on fiddler crabs in hypersaline marshes in Georgia (USA) is likely to due to association defense against avian predators^17^, but had no experimental tests. Bortolus, et al.^16^ also conducted a crab tethering experiment in an Argentinean high marsh, but found no evidence for plant associational defense as a mechanism of plant facilitation on crabs. This may have been due to the fact that their tethering experiments lasted only a few hours and this may not have been long enough to detect predation. Avian predators, even those resident in an area, are often not continuously present and may move around quite a bit. Since predation is potentially high in many physically stressful habitats including the high intertidal^21^,^32^, our work emphasizes the overlooked importance of associational defense in plant-animal facilitations in these habitats.

### Highlighting the importance of facilitation in ecotones

The importance of facilitation in the coastal ecotone demonstrated in our study is consistent with other studies on other types of ecotones, such as steppe-woodland^33^,^34^, alpine treeline^12^,^13^, and open water-lake shore ecotones^35^. Ecotones are often abiotically extreme to species originated from at least one of the adjacent ecosystems^12^ or both (our study). These species are able to persist on ecotones where neighbors ameliorate abiotic stress to their physiological tolerance range, while beyond the ecotones they are limited by abiotic stress. Associational defenses can also be a mechanism driving facilitation on ecotones. For example, ecotones between open water and lake shores provide refugia for fish that would be at risk of predation in open waters or desiccation stress on lake shores^35^. Other mechanisms also likely occur, such as entrapment of propagules^31^. Ecotones have been long known as biodiversity hotspots, and our study together with these previous studies suggest that facilitation is likely one of the key biological forces enhancing diversity in ecotones^36^. Ecotones are often species boundaries sensitive to environmental change, and have been widely used for monitoring the effects of climate change^10^. Future research on facilitation and community organization on species distribution borders or ecotones will be critical to understanding how environmental change affects natural communities.

## Methods

### Study sites

Field work was conducted on the terrestrial borders of two salt marshes in the Yellow River Delta, northern China: Huanghekou (37°43′ N, 119°14′ E) and Yiqian'er (38°05′ N, 118°42′ E). The climate is temperate monsoonal, with cool, dry springs and hot, rainy summers. The long-term annual precipitation is 537.3 mm, and the average temperature is 12.8°C^37^. The terrestrial borders at both sites were bare flats, with scattered *Tamarix* trees and *Suaeda salsa* (Linnaeus) Pallas, an annual succulent. These borders are typically dry and extremely saline (salinities > 100–200 PSU), having a layer of salt on the soil surface.

*Tamarix* is a shrubby recretohalophyte that inhabits coastal and riparian zones in East Asia (native) and North America (invasive^38^). In the Yellow River Delta, *Tamarix* is absent from flooded marsh habitats at lower elevations, but is abundant on the borders between marshes and terrestrial uplands at upper elevations^9^,^39^. *Tamarix* also occurs in terrestrial uplands, but is limited by competition from perennial grasses^9^. *Helice* is a grapsoid crab common in East Asia, and is primarily herbivorous^26^,^40^. The distribution of *Helice* along intertidal gradients in the Yellow River Delta has been previously quantified^41^,^42^. *Helice* occurs in low abundances in flooded mudflats and low marshes at low elevations where other crabs (e.g., *Macrophthalmus japonicas*) are dominant. At higher marshes, *Helice* becomes abundant, being the only common crab species there. On the terrestrial borders at upper elevations, *Helice* occurs only under the canopies of *Tamarix* trees. *Helice* is completely absent from terrestrial uplands beyond tidal influence. Common avian predators include *Larus* spp., *Sterna* spp. and *Ardea* spp.^43^, which forage on the terrestrial borders of salt marshes and nearby water bodies (*Qiang He, personal observation*).

### *Tamarix* removal experiment

To test the hypothesis that removing *Tamarix* would reduce the density of crab burrows, we performed a removal experiment in May 2012. We marked 10 blocks on the terrestrial border at each site. Within each block, we randomly selected two *Tamarix* trees with 2.0–2.5 m^2^ canopies (>3–5 m between trees), and cut down one of the two trees at the soil surface. We established a permanent 1.5 × 1.5 m plot centered at the base of each *Tamarix* tree, and a 1.5 × 1.5 m plot in open areas between trees within each block. Removal treatments were maintained monthly as necessary. We counted number of crab burrows in each plot in May, September 2012 and September 2013 at both sites, and also in May and August 2013 at Huanghekou. We estimated crab abundance by quantifying the density of crab burrows, which followed previous studies from similar habitats^16^,^17^,^25^,^44^. We examined differences in crab burrow density among treatments at each site and sampling date with randomized-blocked ANOVAs followed by Tukey HSD multiple comparisons at the significance level *P* < 0.05. All statistical analysis was done with JMP 10 (SAS Institute, NC, USA).

Edaphic and microclimate conditions were quantified following *Tamarix* removal. Soil cores (5.05 cm diameter × 5 cm depth) were collected in each plot in May and September 2012 and 2013. To determine soil moisture, soil cores were weighed, oven-dried at 60°C for 48 hours, and reweighed. Soil salinity was determined using the initial soil moisture content and the salinity of the water extract from the soil cores (determined using a conductivity meter; model JENCO 3010, Shanghai, China)^45^. Photosynthetically active radiation (PAR), air temperature, air humidity, and soil temperature were quantified at 11:00–13:00 on a cloudless day in early September 2012. PAR, air temperature, and air humidity were quantified 20 cm above the soil surface with quantum light meters (model 3415, Spectrum Technologies, Aurora, IL, USA) and temperature/relative humidity pens (Spectrum Technologies), and soil temperature 5 cm below the soil surface with a soil thermometer (model 6310, Spectrum Technologies)^46^. Differences in edaphic/microclimate factors between treatments on each date were analyzed with randomized-blocked ANOVAs followed by Tukey HSD multiple comparisons. Soil salinity and moisture data were log_10_(*x*)- and (*x*)^3^-transformed, respectively, to increase normality when necessary.

### *Tamarix* simulation experiment

To test the hypothesis that the dependence of *Helice* on *Tamarix* is due to facilitation by shading and predation-protecting, as well as due to food provision, we conducted a shrub simulation experiment crossing shade house and food addition treatments. In May 2012, we located 30 1.5 × 1.5 plots in open areas at Huanghekou, and randomly assigned 6 to each of the five treatments: shade house, food addition, shade house plus food addition, control, and procedural control. Plots assigned to shade house treatments were covered by shade cloth (1.5 × 1.5 × 0.7 m, *l* × *w* × *h*) on all sides. PAR in the shade houses ranged from 100 to 300 μmol/m^2^·s, which was comparable to those under the trees (30–220 μmol/m^2^·s). Shade house treatments also excluded bird access, and no bird footprints/feces were observed within these plots. To allow crab access, shade cloth was 10–15 cm above the soil surface. Procedural controls had shade cloth only on two sides. For food addition treatments, five live 30 cm long *Tamarix* branches were cut, tied at one end, and placed in the center of the plot, and were replaced at least weekly throughout the experiment. We counted the number of crab burrows in each plot in mid-September. The experiment was discontinued in the following year, due to potential winter ice and human damage. We examined the separate and interactive effects of shade house and food addition on number of crab burrows (sqrt-transformed) with a two-way ANOVA, and differences between control and procedural control treatments with a Wilcoxon test.

To quantify the effects of shade treatments on edaphic and microclimate conditions, we determined soil salinity, soil moisture, PAR, air temperature, air humidity, and soil temperature in each plot in September, using the same methods as described above. We examined differences in each factor between treatments using nonparametric multiple comparisons for air temperature data (Dunn method for joint ranks; the air temperature data did not meet the normality assumptions of parametric tests) and Tukey HSD multiple comparisons for all others.

### Crab tethering experiment

To test the hypothesis that *Tamarix*'s alleviation of both abiotic stress and predation is critical to the fate of *Helice* on the terrestrial borders of salt marshes, we conducted a crab tethering experiment. In August 2012, we tethered *Helice* (carapace width 24–29 mm; hand collected in the field) on the terrestrial border at Huanghekou. We placed a tethered crab in the center of each of 24 open area plots, 12 *Tamarix* plots and the 12 shaded plots used in the shrub simulation experiments. Crabs in half of the open area plots were protected from predation with cages (25 × 25 × 20 cm) of galvanized-steel hardware cloth (7 mm mesh size). Tethers were constructed of 15 cm long fishing line, tied around the carapace of crabs, secured to the carapace with cyanoacrylic glue, and held by steel stakes pushed flush with the soil surface^16^. We deployed *Helice* at dusk, and examined their survivorship after 24 hours. Crabs that died from either desiccation stress (with intact body) or predation (with dismembered body and presence of bird footprints) were counted. Treatment differences in survivorship were analyzed with chi-square tests (Fisher's exact test).

## Author Contributions

Q.H. and B.C. designed the study, Q.H. performed the experiments and analyzed the data, and Q.H. and B.C. wrote the paper.

## Supplementary Material

Supplementary Information Figure S1
