# Supplementary material for: Multiple mechanisms sustain a plant-animal facilitation on a coastal ecotone
Source: Sci Rep. 2015 Feb 27;5:8612. doi: 10.1038/srep08612 (PMC4342566; doi:10.1038/srep08612)
Supplement: Supplementary Information — Figure S1 [file srep08612-s1.pdf]

Supplementary information for

Multiple mechanisms sustain a plant-animal facilitation on a coastal ecotone

Qiang He, Baoshan Cui*

School of Environment, State Key Laboratory of Water Environment Simulation, Beijing Normal University, Beijing 100875, China

* Corresponding author. Email: cuibs@bnu.edu.cn

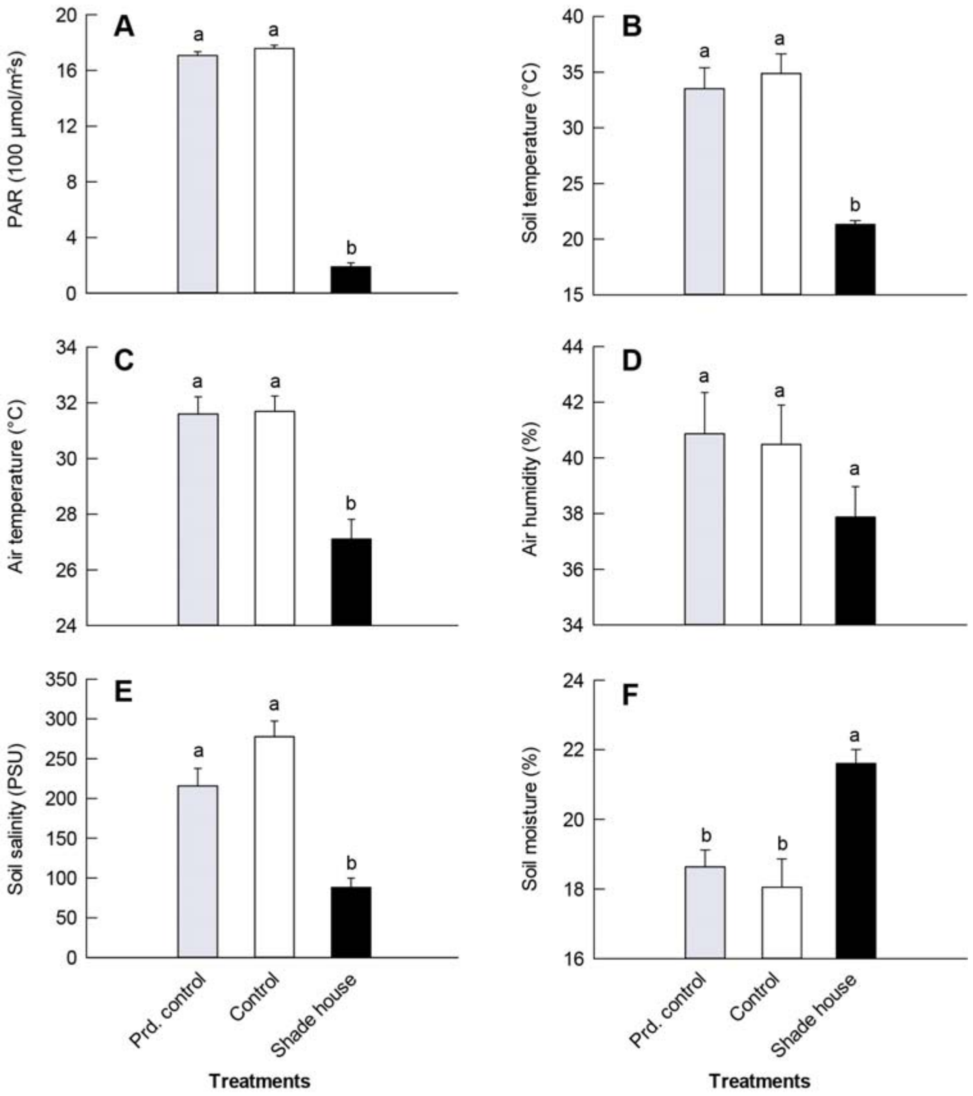

Fig. S1 *Tamarix* simulation experiment: microclimate and soil factors in each treatment. A, Photosynthetically active radiation (PAR); B, soil temperature; C, air temperature; D, air humidity; E, soil salinity; and F, soil moisture. Data are means + SE (n = 6). Prd. control indicates procedural control. Bars sharing a letter are not significantly different from one another ($P > 0.05$; Dunn Joint Ranks for air temperature, and Tukey HSD multiple comparisons for all others).
